# Effect of curing conditions on heat resistance in white chocolate

**DOI:** 10.1002/fsn3.4292

**Published:** 2024-07-09

**Authors:** Julie A. Laughter, B. Douglas Brown, Ramaswamy C. Anantheswaran

**Affiliations:** ^1^ Department of Food Science Pennsylvania State University University Park Pennsylvania USA; ^2^ Technical Center The Hershey Company Hershey Pennsylvania USA

**Keywords:** curing, hardness, heat resistance, moisture content, SEM, white chocolate

## Abstract

Heat‐resistant chocolate is of much interest to confectionery companies for marketing in tropical areas. Methods exist to produce heat‐resistant chocolate by exposing the product to high relative humidity (RH) and increased temperatures. The overall objective of this research project was to explore the curing of white chocolate (30% whole milk powder, 44% sucrose, and 26% cocoa butter) to make it heat resistant and able to be picked up at 33 and 55°C. The curing involved storing solid chocolate samples at 83% RH at 29°C for 1 week. Moisture content before and after curing was measured using the Karl Fischer technique. Force required to penetrate (hardness) was measured at 29°C using a texturometer. Curing samples of white chocolate bars at a lower relative humidity (50% RH at 18°C for 12 weeks or 50% RH at 29°C for 12 weeks) also resulted in a heat‐resistant chocolate that exhibited internal structure, as monitored by SEM. The moisture content in heat‐resistant milk chocolate increased from an average value of 0.84% to 4.6% during the curing process. Curing increased the penetration force, indicating the development of internal structure. This study showed that heat resistance in white chocolate can be achieved by curing solid chocolate samples at controlled humidity and temperature conditions.

## Graphical abstract

The overall objective of this research project was to explore curing conditions to make heat‐resistant white chocolate. The curing in this study involved storing solid chocolate samples at three combinations of temperature, relative humidity and exposure time. Curing resulted in a heat‐resistant chocolate that could be picked up even at a temperature of 55°C.

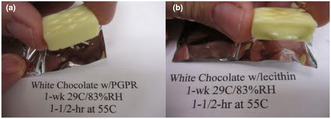

## 1 INTRODUCTION

Chocolate is a dispersion of solids in a continuous, semi‐crystalline fat phase of cocoa butter. The main types of chocolate are milk, dark, and white. Milk chocolate is a suspension of cocoa solids, milk solids, and sugar in cocoa butter. White chocolate also has milk solids and sugar but does not contain nonfat cocoa solids, while dark chocolate includes cocoa solids and sugar but no or very little milk ingredients. The melting profile of chocolate is determined by the fatty phase, most of which is composed of cocoa butter. As the temperature approaches 28–32°C, chocolate begins to soften and lose shape, finally melting at temperatures between 34 and 36°C. At its melting point, chocolate adheres to wrappers and sticks to hands (Beckett, 2008).

In tropical countries, development of heat‐resistant chocolate will better meet consumer demands. Heat‐resistant chocolate should resist adhering to wrappers at temperatures exceeding 30°C, maintain shape at temperatures above 35°C and not stick to hands at 40°C (Beckett, 2008; Best et al., 2007; Schenk & Peschar, 2004). The finished product should have a texture and flavor comparable to that of regular chocolate (Best et al., 2007).

Heat‐resistant chocolate has been referred within the literature as shape‐sustaining chocolate (Beckett, 1995; Kempf, 1958; Kempf & Downey, 1956; Nalur & Napolitano, 2002; O'Rourke, 1959). It has also been defined as chocolate that does not adhere to the wrapper at temperatures exceeding 30°C (Schubiger & Rostagno, 1965); it will maintain its shape when exposed to temperatures above 35°C (Alander et al., 1996); it is not sticky to the direct touch at 40°C (Takemori et al., 1992); it will remain stiff at 50°C (Giddey & Dove, 1984).

Many patents describe methods to produce heat‐resistant chocolate, including exposing chocolate to high relative humidity (RH) and high temperatures (Kempf, 1958; O'Rourke, 1959). We can induce heat resistance in chocolate by creating inherent structure formed through an edifice of sugar particles (Schubiger & Rostagno, 1965). Stortz and Marangoni (2011) described compositional parameters and process parameters that would create structure in chocolate. Inherent structure can be induced by adding water, monosaccharides, amorphous sugars, polyols, fiber, starch, or protein can create heat resistance in chocolate (Afoakwa et al., 2007; Finkel, 1990; Friedman, 1921; Killian & Coupland, 2012; Kruger & Freund, 2001; Lopez et al., 2010). The combination of high relative humidity and increased temperatures can promote the formation of heat‐resistant structure in chocolate (Kempf & Downey, 1956; O'Rourke, 1959).

Dicolla et al. (2019) developed a sensory method to describe heat‐resistant chocolate. Using a trained sensory panel, 13 sensory attributes (firmness to touch, stickiness to fingers, snap, abrasiveness, hardness with incisors, fracturability, cohesiveness of mass, time to melt, firmness with tongue, adhesiveness to teeth, number of particles, oily mouthcoating, and chocolate messiness) were identified as sensory attributes of heat‐resistant chocolate.

The overall objective of this research project was to explore curing of white chocolate to make it heat resistant and able to be picked up at 33 and 55°C.

## 2 MATERIALS AND METHODS

### 2.1 Materials

Sucrose was obtained from Domino Sugar (Arabi, LA). Whole milk powder (WMP) was made by spray‐drying and sourced from Land O′ Lakes (Carlisle, PA). Cocoa butter, lecithin, and polyglycerol polyricinoleate (PGPR) were provided by the Hershey Company (Hershey, PA).

### 2.2 Manufacture of white chocolate

White chocolate (WC) production began with initial mixing of sugar, nonfat milk solids, and cocoa butter in a Hobart 12‐qt. bowl and mixer. Formulas of the various white chocolate samples made are listed in Table 1. Heat was applied during mixing until the temperature of the chocolate was 40 ± 3°C. After blending the ingredients, the sample was double refined using a Buhler 3‐roll refiner to achieve a particle size of 18 ± 2 μm.

**TABLE 1:** White chocolate formulas.

Ingredients	WC with lecithin	WC with PGPR	WC without emulsifier
WMP	30%	30%	30%
Sucrose	44%	44%	44%
Cocoa butter	25.7%	25.9%	26%
Lecithin	0.3%		
PGPR		0.1% blending	

Particle size of the refined flake was measured by dispersing the sample in mineral oil and using a handheld Mitutoyo IP 65 micrometer (Mitutoyo Corporation, Kawasaki, Japan). The refined flake was then mixed with cocoa butter so the total fat in the chocolate mass was 26%–28%. Conching was accomplished in a Hobart 12‐qt. bowl and mixer for a duration of 3 h at 50°C. The sample was then standardized by adding the remaining cocoa butter and lecithin or polyglycerol polyricinoleate (PGPR) (Laughter, 2012).

White chocolate was made with whole milk powder (WMP) and different amounts of emulsifiers as shown in Table 1. Samples were tempered using Sinsation K5 tempering machines (Chocovision, Poughkeepsie, NY). The standardized chocolate was placed in the tempering machine where it was allowed to heat to 40°C. During this time, the seed was made by spreading a thin layer of chocolate onto a marble table at 21°C. The seed was added once the chocolate sample was cooled to 31±1°C. The temper of the chocolate was verified using a temper meter (Tricor Systems Inc., Elgin, IL).

Chocolate was then poured into tablet molds on a vibrating table to remove air bubbles. The bars were placed in a cooler at 10°C for 15 to 30 min. Once set, the bars were taken out of the molds and packaged in heat‐sealed aluminum moisture impermeable bags. The bars were stored at 18°C until further testing.

### 2.3 Curing conditions

Chocolate samples were stored at controlled humidity and temperature conditions for various durations (hereafter referred to as curing) to accelerate the formation of structure (Dicolla, 2009; Dicolla et al., 2019). The curing involved storing solid chocolate samples in an environmental chamber at controlled humidity (at 50% RH) and temperature (18 or 29°C) for 12‐week duration.

To expedite the curing process, a higher humidity of 83% RH was used. The chocolate bars were placed into a jar containing 83% RH KCl salt slush at 29°C for 7 days (Greenspan, 1977). The KCl salt slush provided a suitable relative humidity environment to promote heat‐resistant structure formation without causing deliquescence of sucrose, which occurs at ~85% RH and 25°C (Listiohadi et al., 2005). Small crystals of thymol were placed in the curing jars to inhibit mold growth.

### 2.4 Moisture content measurement

The Karl Fischer method (784 KFP Titrino titrator with a 703 Titration stand, Brinkmann, Westbury, NY) was used to measure the moisture content of the samples before and after curing.

### 2.5 Texturometer measurement

Stable Micro Systems Texture Analyzer TA‐XT2 (Surrey, UK) and the software Texture Exponent 32 (Stable Micro Systems, Surrey, UK) were used to measure the amount of force required to penetrate pre‐cured and post‐cured white chocolate samples. The instrument was calibrated for force and probe position. Molded chocolate samples (9 cm × 3 cm × 1.5 cm) were placed on the sample stage underneath a 2‐mm‐diameter blunt stainless steel probe. The hardness of the samples before and after curing was measured at 29^o^C with the texturometer. The maximum force required to penetrate the sample 7 mm from the surface in the texturometer was reported as hardness values before and after curing (Laughter, 2012).

### 2.6 Scanning electron microscopy (SEM)

The samples were defatted using nonpolar solvent (Schubiger & Rostagno, 1965). The nonpolar solvent we used was hexane, and it dissolved the solid fat in the chocolate matrix leaving behind any structural residual. After 24 h of immersion in hexane, the defatted chocolate sample was sputter‐coated with gold and placed into the vacuum chamber. Images were performed at full vacuum and 8 kV at various magnifications. A Philips SEM (Portland, OR) was used to examine the structure of heat‐resistant chocolate.

### 2.7 Statistical data analysis

Data on fat content, moisture content, and hardness were analyzed using one‐way ANOVA and Tukey's post hoc test. The moisture content of individual ingredients was analyzed using paired *t*‐tests. All measurements were made in triplicate, and all statistics were performed using MINITAB Student Release 14 (version 14.11.1, State College, PA).

## 3 RESULTS AND DISCUSSION

### 3.1 Moisture content measurement

Moisture content is important for the development of heat‐resistant structure in chocolate. The initial moisture content between the various samples was not significantly different before curing (Table 2). However, after curing, all of the samples showed a significant (*p* < .05) increase in moisture content due to the high relative humidity of the curing environment. The WMP chocolate with PGPR had the highest moisture content after curing.

**TABLE 2:** Moisture content^1^ of chocolate samples (made with WMP and with different emulsifiers) before and after curing at 83% RH and 29°C for 1 week.

Chocolate sample	Precured moisture content	Postcured moisture content
	(g moisture/100 g wet basis)	(g moisture/100 g wet basis)
WMP with lecithin	0.92 ± 0.086^a^	4.6 ± 0.11^b^
WMP with PGPR	0.77 ± 0.067^a^	5.1 ± 0.21^c^
WMP with no added emulsifier	0.82 ± 0.038^a^	4.2 ± 0.31^b^
Average	0.837	4.63

^1^ Data are presented as average values ± standard deviation.

^a,b,c^ Same letter implies no significant difference at *α* = .05.

Ghosh et al. (2002) stated that the rate of moisture migration is affected by several factors, including the chemical potential gradient between areas with higher water activity to those with lower water activity. Temperature also influences moisture migration. Biquet and Labuza (1988) observed that raising the temperature from 20 to 26°C tripled the water vapor transmission rate in dark chocolate films, while increasing the temperature from 10 to 20°C did not significantly affect this rate. They postulated that the temperature increase from 20 to 26°C increased the liquid fat content, making moisture migration easier throughout the dark chocolate films.

### 3.2 Texture measurement

The texturometer data between the various samples were not significantly different (*p* > .05) before curing (Table 3). However, after curing, all of the samples showed a significant increase in hardness, possibly due to creation of internal structure.

**TABLE 3:** Average penetration force^1^ (g) of chocolate samples before and after curing at 83% RH and 29°C for 1 week.

Samples	Precured average max force (g)	Postcured average max force (g)
WMP with lecithin	240 ± 19^a^	300 ± 23^b^
WMP with PGPR	260 ± 37^ab^	380 ± 28^c^
WMP with no added emulsifier	270 ± 37^ab^	420 ± 81^c^
Average	257	367

^1^ Data are presented as average values ± standard deviation.

^a,b,c^ Same letter implies no significant difference at *α* = .05.

During curing, the samples were exposed to conditions favorable for potential lactose crystallization as reported by Ziegleder et al. (2004). Crystallization of lactose in the chocolate can lead to a thickening of the paste caused by the interparticle bridging between lactose molecules.

Bars with PGPR required significantly more force than those made with lecithin. It has been reported that lecithin and PGPR behave in different manners when added to sugar dispersion in a fat matrix (Garbolino, 2002). The polar heads of lecithin molecules adsorb to the surface of sugars in a multilayer domain. The polar portions of PGPR also adsorb to the surface of hydrophilic ingredients, but these polar parts are interspersed along the highly branched PGPR molecule. Instead of a multilayer domain, the absence of a polar head causes the hydrophilic groups on PGPR to form a loosely bound film around sugar molecules while the hydrophobic portions protrude into the fatty phase (Garbolino, 2002).

While both lecithin and PGPR have water‐binding properties, lecithin creates a more compact structure surrounding sugar molecules. Moisture can travel through the reverse micelles and multilayer system, but this complex matrix could obstruct moisture from reaching the hydrophilic ingredients.

### 3.3 Handling of heat‐resistant chocolate

Dicolla (2009) categorized temperature tolerance of chocolate a mild, intermediate, and extreme heat resistance, with extreme heat resistance, referring to chocolate that is able to withstand temperatures exceeding 37.9°C. To characterize heat resistance, cured white chocolate samples containing either PGPR or lecithin were exposed to various temperatures and time intervals.

**FIGURE 1:**
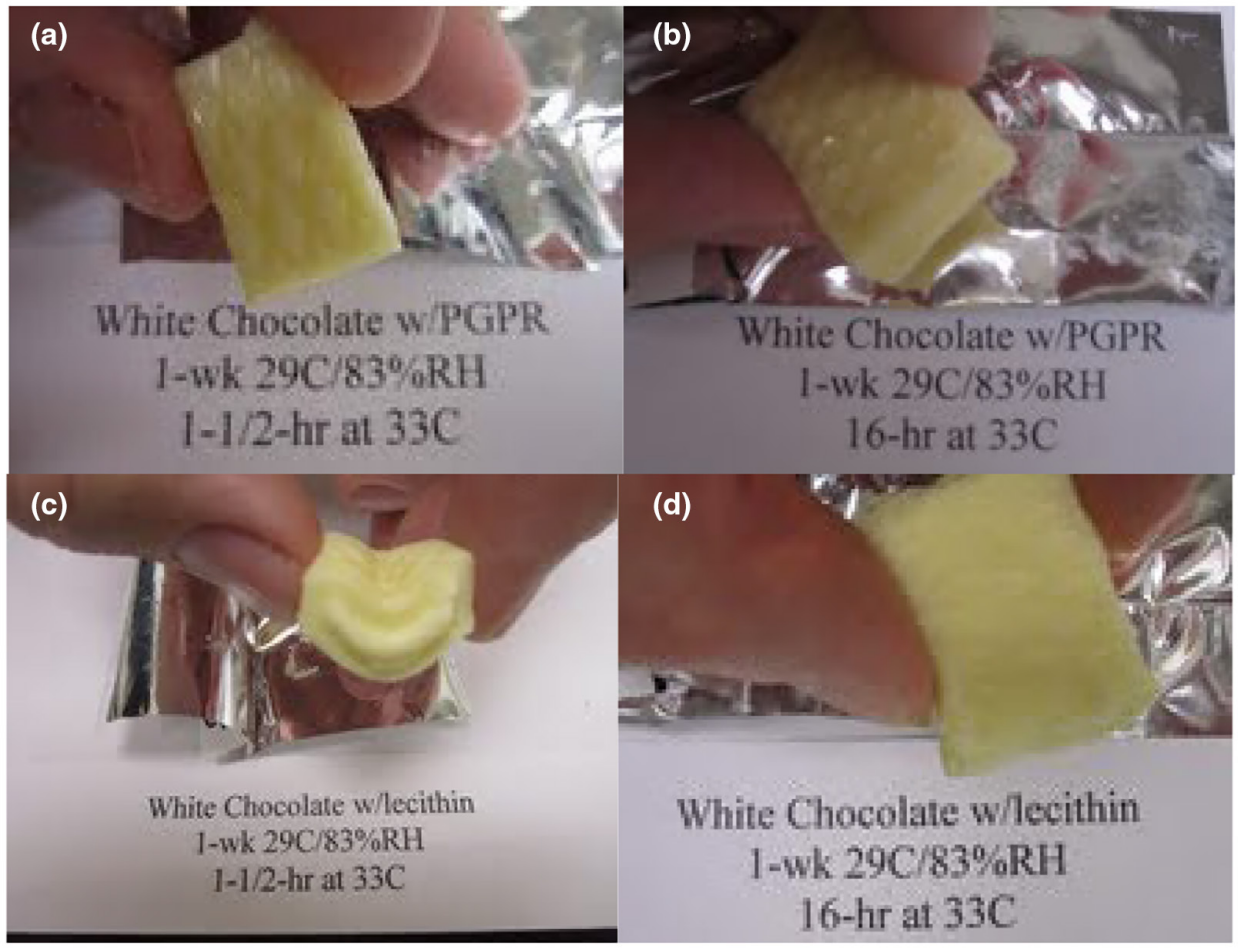
Post‐cured (1 week at 29°C and 83% RH) heat‐resistant white chocolate handleability after exposure to 33°C for various time intervals: (a) PGPR sample after 1.5 h, (b) PGPR sample after 16 h, (c) lecithin sample after 1.5 h, and (d) lecithin sample after 16 h.

**FIGURE 2:**
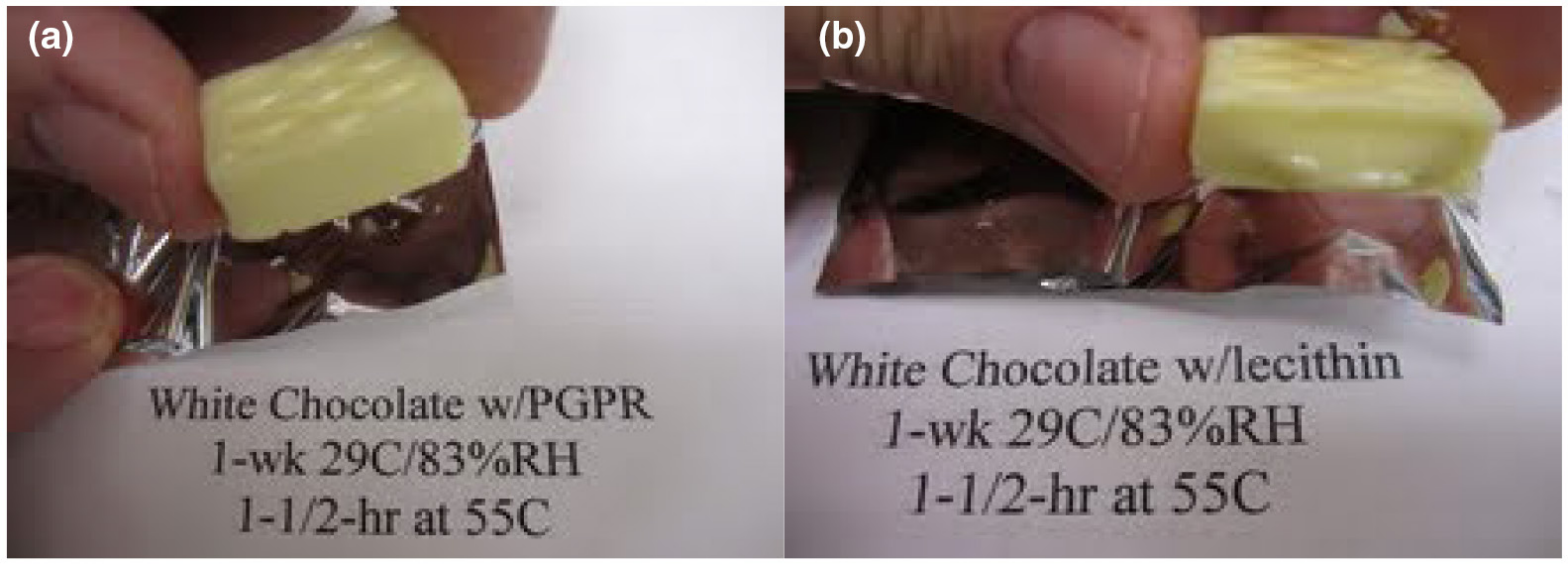
Post‐cured (1 week at 29°C and 83% RH) heat‐resistant white chocolate handleability after exposure to 55°C for 1.5 h: (a) PGPR sample, (b) lecithin sample.

Cured samples with PGPR or lecithin maintained their shape and were able to be picked up after exposure to 33°C for up to 16 h (Figure 1). Cured samples with PGPR or lecithin maintained their shape and were able to be picked up even after exposure to 55°C for 1.5 h (Figure 2). Uncured bars melted at these same elevated temperature and time conditions, and they could not be picked up.

Other curing conditions of 19°C at 50% RH for 12 weeks and 29°C at 50% RH for 12 weeks also resulted in chocolate that was heat resistant to 55°C (Laughter, 2012).

### 3.4 SEM analysis

**FIGURE 3:**
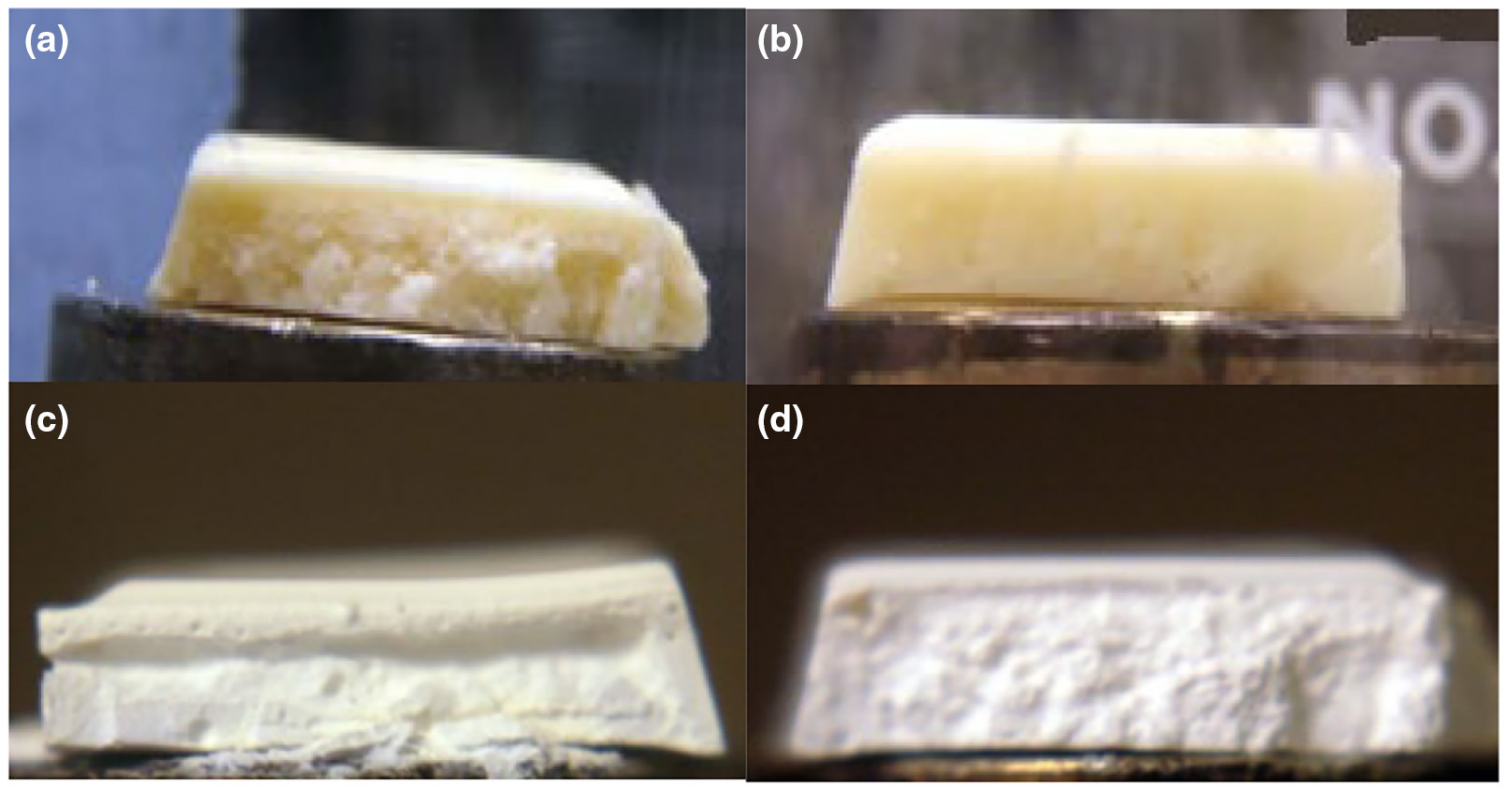
Fifteen‐mm‐thick white chocolate samples cured for 12 weeks at 18°C/50% RH: (a) before hexane immersion, (c) after hexane immersion. Ten‐mm‐thick white chocolate cured for 12 weeks at 29°C/50% RH: (b) before hexane immersion, (d) after hexane immersion.

After curing chocolate at 18°C/50% RH or 29°C/50% RH for 12 weeks, samples were immersed in hexane for 24 h. These samples exhibited a nonfat structure, indicating that both curing conditions could create a heat‐resistant white chocolate (Figure 3). Chocolate cured at 18°C/50% RH showed a slight collapse in the middle as compared to samples cured at 29°C/50% RH. Without curing, the white chocolate completely collapsed to a powder after hexane treatment.

While curing at 29°C/50% RH for 12 weeks was sufficient in creating an internal structure, the effect of increasing relative humidity to 83% on the rate of curing was investigated. Increasing the relative humidity from 50% to 83% shortened the curing to 1 week, and we could pick up the sample of chocolate (Figure 1).

**FIGURE 4:**
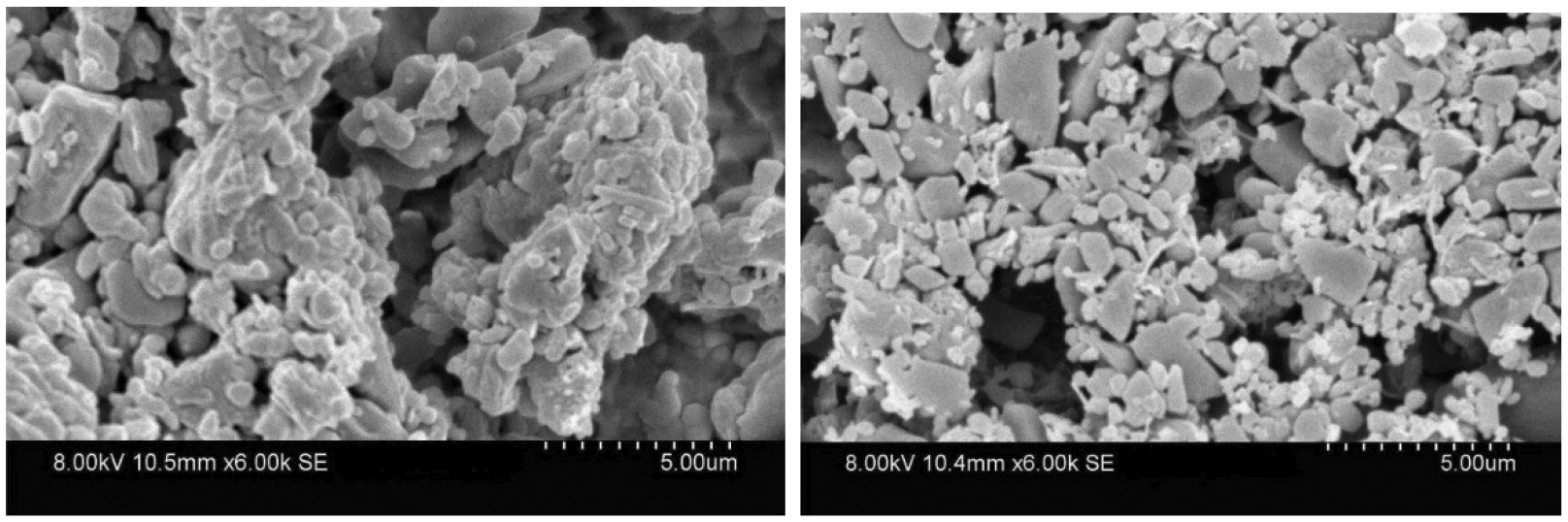
Two SEM views of white chocolate samples cured at 29°C/50% RH for 12 weeks and hexane immersed (each tic mark represents 0.5 μm).

The defatted structure of chocolate sample cured at 29°C/50% RH for 12 weeks was further examined using SEM. The SEM images showed interconnected particles (Figure 4), but the mechanism by which they were connected is not very apparent.

**FIGURE 5:**
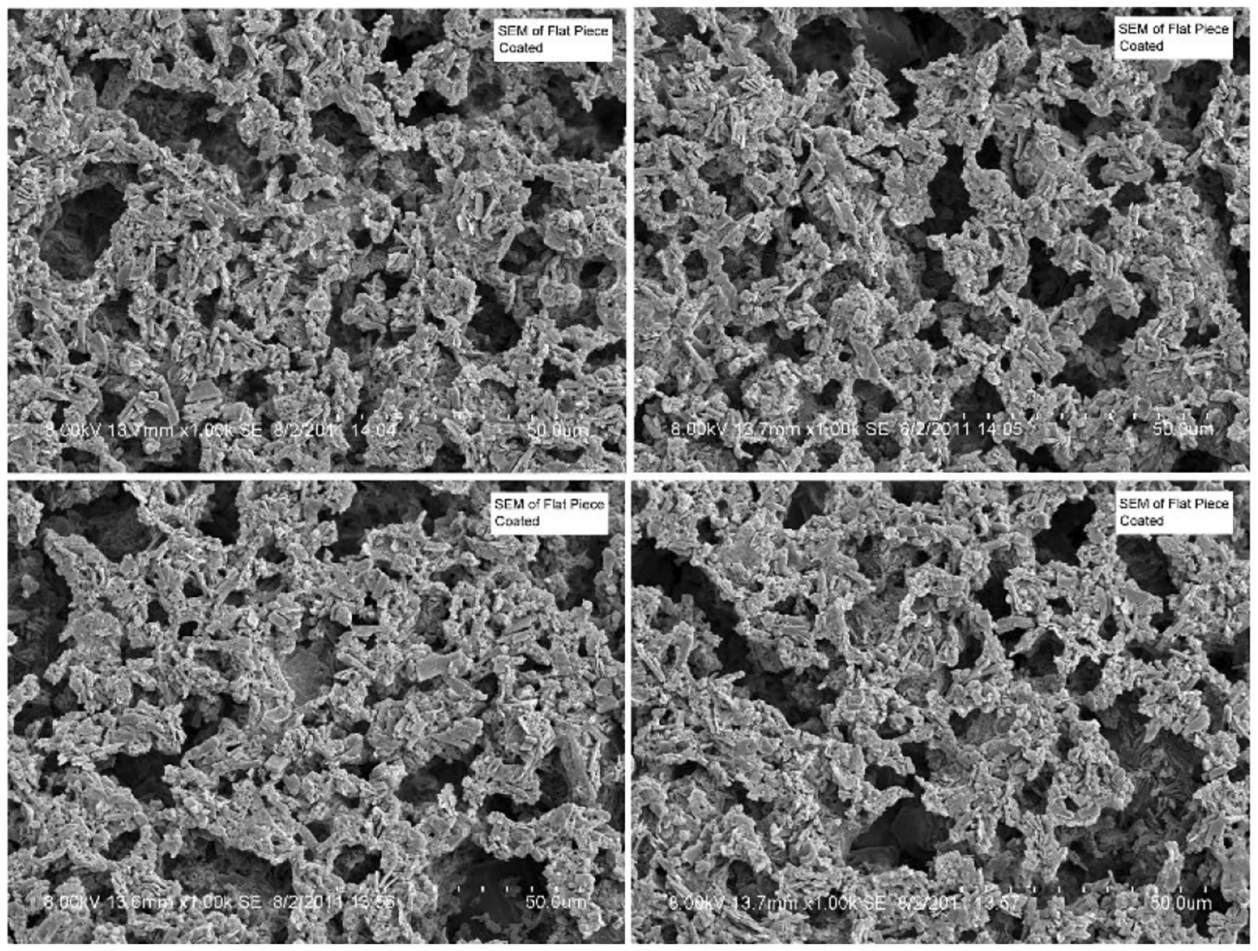
Four SEM views of white chocolate samples with 0.3% lecithin and cured at 29°C/83% RH for 7 days (each tic mark represents 5 μm).

White chocolate samples were also made with 0.3% lecithin, 0.1% PGPR, or no added emulsifier and were cured at 29°C/83% RH for 1 week. All cured samples exhibited structure after 24 h in hexane immersion, and SEM images of samples with 0.3% lecithin showed an interconnected structure (Figure 5).

It was further hypothesized that less time would be needed to complete the curing process of a thinner sample. A screening study was conducted to determine the minimum time needed for curing a 1.5‐mm‐thick white chocolate sample. Chocolate was poured into a 1.3‐cm tall and 4.4‐cm‐diameter cup to create a 1.5‐mm‐thick layer and was cured for different lengths of time. The chocolate sample cured for 3 h at 29°C and 83% RH after hexane immersion had no structure, whereas the sample cured for 4 h at 29°C and 83% RH showed internal structure and could be removed from the sample cup.

## 4 CONCLUSIONS

The overall objective of this research project was to explore curing conditions to make heat‐resistant white chocolate. The curing in this study involved storing solid chocolate samples at 83% RH at 29°C for 1 week. Curing samples of chocolate at a lower relative humidity (50% RH at 18°C for 12 weeks or 50% RH at 29°C for 12 weeks) also resulted in a heat‐resistant chocolate that could be picked up at 33 and 55°C and exhibited internal structure by SEM. Curing increased the moisture content and the force to penetrate the chocolate samples, indicating the development of internal structure. Curing resulted in a heat‐resistant chocolate that could be picked up even at a temperature of 55°C.

## AUTHOR CONTRIBUTIONS

**Julie A. Laughter:** Conceptualization (equal); data curation (equal); formal analysis (equal); investigation (equal); methodology (equal); validation (equal); visualization (equal). **Ramaswamy C. Anantheswaran:** Conceptualization (equal); data curation (equal); formal analysis (equal); investigation (equal); methodology (equal); project administration (equal); supervision (equal); validation (equal); visualization (equal); writing – original draft (equal); writing – review and editing (equal). **B. Douglas Brown:** Conceptualization (equal); data curation (equal); formal analysis (equal); investigation (equal); methodology (equal); project administration (equal); supervision (equal); validation (equal); visualization (equal); writing – original draft (equal); writing – review and editing (equal).

## CONFLICT OF INTEREST STATEMENT

The authors declare that they do not have any conflict of interest.

## ETHICS STATEMENT

This study does not involve any human or animal testing.

## Data Availability

The authors confirm that the data supporting the findings of this study are available within the article. Supplementary materials are availale in the Masters thesis (Laughter, J. A. 2012), which is referenced in the article.
